# A description of physical therapists' knowledge in managing musculoskeletal conditions

**DOI:** 10.1186/1471-2474-6-32

**Published:** 2005-06-17

**Authors:** John D Childs, Julie M Whitman, Phillip S Sizer, Maria L Pugia, Timothy W Flynn, Anthony Delitto

**Affiliations:** 1 US Army-Baylor University Doctoral Program in Physical Therapy, Fort Sam Houston, San Antonio, TX, USA; 2 Department of Physical Therapy, Regis University, Denver, CO, USA; 3 Department of Physical Therapy, Texas Tech University, Lubbock, TX, USA; 4 Department of Physical Therapy, Los Angeles Air Force Base, Los Angeles, CA, USA; 5 Department of Physical Therapy, University of Pittsburgh, Pittsburgh, PA, USA

## Abstract

**Background:**

Physical therapists increasingly provide direct access services to patients with musculoskeletal conditions, and growing evidence supports the cost-effectiveness of this mode of healthcare delivery. However, further evidence is needed to determine if physical therapists have the requisite knowledge necessary to manage musculoskeletal conditions. Therefore, the purpose of this study was to describe physical therapists' knowledge in managing musculoskeletal conditions.

**Methods:**

This study utilized a cross-sectional design in which 174 physical therapist students from randomly selected educational programs and 182 experienced physical therapists completed a standardized examination assessing knowledge in managing musculoskeletal conditions. This same examination has been previously been used to assess knowledge in musculoskeletal medicine among medical students, physician interns and residents, and across a variety of physician specialties.

**Results:**

Experienced physical therapists had higher levels of knowledge in managing musculoskeletal conditions than medical students, physician interns and residents, and all physician specialists except for orthopaedists. Physical therapist students enrolled in doctoral degree educational programs achieved significantly higher scores than their peers enrolled in master's degree programs. Furthermore, experienced physical therapists who were board-certified in orthopaedic or sports physical therapy achieved significantly higher scores and passing rates than their non board-certified colleagues.

**Conclusion:**

The results of this study may have implications for health and public policy decisions regarding the suitability of utilizing physical therapists to provide direct access care for patients with musculoskeletal conditions.

## Background

Musculoskeletal conditions account for roughly 25% of patient complaints in the primary care setting[1]. However, physicians have been shown to lack confidence in their evaluation and treatment skills of these patients [2-6]. Although its de-emphasis in medical school curricula has been repeatedly implicated,[1,5,7-9] almost half of American medical schools still do not require any formal training in musculoskeletal medicine[10]. This lack of confidence is reflected by poor performance on formal assessments of knowledge in musculoskeletal medicine[7] and less than optimal practice patterns for patients with musculoskeletal conditions[11]. Freedman and Bernstein[7] assessed knowledge in musculoskeletal medicine among 85 physicians during their first week of their internship following graduation from medical school using a standardized examination. The mean score was just under 60%, with only 18% of physicians scoring above a level determined orthopaedic program directors as the minimum threshold necessary to establish competency in musculoskeletal medicine in the primary care setting[7]. Matzkin et al[12] recently demonstrated similar suboptimal levels of knowledge in musculoskeletal medicine among medical students and residents. Except for orthopaedists, they also found that experienced physicians across a variety of specialties demonstrated less than adequate knowledge related to musculoskeletal medicine. The authors concluded that training in both medical school and non-orthopaedic residency training programs was inadequate, a sentiment that has been echoed elsewhere[13].

Considerable evidence supports the benefits of early access to physical therapy care [14-18]. In particular, physical therapists increasingly provide their services without physician referral (ie, direct access). Seventy percent of the public reports they would seek care from a physical therapist without physician referral for musculoskeletal conditions, [19] with 39 states having passed legislation supporting this mode of healthcare delivery [20]. Multiple studies have demonstrated that physical therapists can provide safe and cost-effective care for patients with musculoskeletal conditions in direct access practice settings, supporting the expansion of direct access physical therapy services [21-26]. For example, physician referral episodes of care reportedly increased physical therapy claims by 67%, office visits by 60%, and costs by 123% than when patients directly accessed physical therapy without physician referral[24].

Despite the curricular emphasis placed on the management of musculoskeletal conditions in physical therapy programs, to date few studies have described physical therapists' knowledge of the skills necessary to manage these patients in a direct access setting. A musculoskeletal written examination has been developed and validated for this purpose[7,27]. The examination has been administered to physician interns,[7] medical students and residents,[12] and a variety of physician specialists,[12] making it a pragmatic reference standard for the initial assessment of knowledge in managing musculoskeletal conditions among physical therapist students and licensed physical therapists. Therefore, the purpose of this study was to describe physical therapists' knowledge in managing musculoskeletal conditions using this examination. These data combined with clinical studies demonstrating the benefits of direct access physical therapy [21-26] may further clarify the role of physical therapists in direct access environments.

## Methods

We used a cross-sectional design to describe knowledge in managing musculoskeletal conditions among physical therapist students and licensed physical therapists in the uniformed services. The study was approved by the Institutional Review Boards at Wilford Hall Medical Center in San Antonio, TX and at Texas Tech University in Lubbock, TX before subject recruitment and data collection began. All subjects provided informed consent prior to participation.

Based on an *a priori *sample size estimation, a total of 26 first-professional physical therapy programs accredited by the Commission for Accreditation of Physical Therapy Education were randomly selected for participation. Educational programs are rapidly transitioning to doctoral programs, with approximately 80% of programs having completed the transition to the Doctor of Physical Therapy degree or in the transitioning process at the time of the study [28]. Therefore, randomization was blocked by the degree to be conferred upon graduation: master's (n = 13) vs. doctoral (n = 13) to permit comparisons based on degree status. Program directors were contacted initially by email to inform them of their program's selection, describe the study procedures, and invite the program's participation. Students in these programs were in the terminal phase of their program's curriculum, defined as having completed substantial portions of the didactic curriculum and clinical affiliations. All licensed physical therapists in the four uniformed health services (U.S. Air Force, U.S. Army, U.S. Navy, and U.S. Public Health Service) with at least one year of clinical experience were also invited to participate.

Participants completed the identical examination originally developed by Freedman and Bernstein to assess knowledge in musculoskeletal medicine among physician interns, [7] and more recently administered to medical students, residents, and a variety of physician specialists [12]. The examination consists of 25 open-ended questions that were selected based on commonly encountered musculoskeletal diagnoses encountered in the primary care setting (ie, fractures and dislocations, low back pain, sciatica, and arthritis) and consideration of orthopaedic emergencies that warrant immediate referral to an orthopaedic surgeon or the emergency department (ie, compartment syndrome, hip dislocation, etc.) [7]. Additional details related to the development and validation of the examination are reported elsewhere [7,27].

The examination was administered in a web-based format using Web Surveyor, version 3.6 (Web Surveyor Corporation, Herndon, VA). No time limit was imposed to be consistent with previous methodology [7,12]. Subject confidentiality was strictly maintained through assignment of a unique computer-generated code. Administration of the examination was preceded by a brief demographic survey that queried patients as to their educational background, board-certification status (Orthopaedic and/or Sports Clinical Specialist designation via the American Board of Physical Therapy Specialties), experience in different practice settings, and familiarity with the studies by Freedman and Bernstein [7,27]. Data from any therapists familiar with the studies by Freedman and Bernstein [7,27] were excluded from analysis because the examination questions and answer key were published verbatim in these manuscripts.

Educational programs were requested to have participants complete the study in a group setting with a proctor present (eg, a computer lab) when possible. This would insure that participants did not use any outside resources (ie, textbooks, information available on the internet, personal communication, etc.) to assist them in answering the questions. To maximize participation, however, programs were alternatively given the option to have participants complete the study on their own if a computer lab or similar arrangement was unavailable, or if a participant was not available at the designated time. Licensed physical therapists were also asked to complete the study in a proctored setting. All participants were queried at the end of the study as to whether they used any outside resources to assist them in the completion of the examination. The results of the demographic survey and content of the examination were stored in a secure, password-protected centralized database for subsequent analysis.

### Data analysis

A total of 6 judges, blinded to the demographic survey results and whether the participant was a physical therapist student or licensed physical therapist scored blocks of 4–6 questions, resulting in each question being scored by two raters. Judges were physical therapist faculty with considerable experience in providing direct access care for patients with musculoskeletal conditions. Each rater was also trained in the scoring procedures by one of the investigators. An overall score and passing rate were determined using identical procedures as those described by Freedman and Bernstein,[7] however a brief review is provided here. Each question was assigned a maximum possible of 1 point. Partial credit was assigned based on the criteria for partial credit outlined in the answer key [7]. Scores were not penalized for incorrect spelling. Sums of individual scores represented the overall score, which was multiplied by 4 to obtain a percentage score. Inter-rater reliability of the overall score was examined using the intraclass correlation coefficient (ICC), equation 3,1 [29]. The ICC and associated 95% confidence interval was 0.91 (0.89, 0.92). Given a sufficiently high reliability coefficient, only data from the first rater were used in the analysis. Using the results from a single rater is also consistent with the procedures utilized by Freedman and Bernstein [7]. Participants were judged to have passed the examination if their score exceeded the previously established threshold of 73.1% [7].

Descriptive statistics, including frequency counts for categorical variables and measures of central tendency and dispersion for continuous variables were calculated to summarize the data using SPSS for Windows 11.0.1 (Chicago, IL). Independent sample t-tests were used to directly compare differences in knowledge between educational programs conferring the doctoral versus master's degree and between licensed physical therapists who were board-certified and those who were not. Differences in the passing rates among the physical therapist subgroups were examined using the Pearson chi-square statistic. The alpha-level was established *a priori *to be 0.05 utilizing a two-tailed test.

## Results

174 physical therapist students across 12 out of the 26 (46%) randomly selected programs volunteered and completed the study, representing 52% of students within these programs. The mean age of physical therapist student participants was 26.7 (3.3) (range = 22–40). Ninety-two percent of physical therapist student participants (n = 160) completed the study in a proctored setting. 63.8% of physical therapist students (n = 111) were enrolled in doctoral degree programs. 182 licensed physical therapists in the uniformed services completed the study, representing 44% of uniformed physical therapists. The mean age of the licensed physical therapist participants was 37.7 (6.7) (range = 25–55). The average years of experience was 8.7 (6.3) (range = 1–30) and 28.6% (n = 52) of licensed physical therapists were board-certified.

No participant reported having received assistance to complete the examination. One licensed physical therapist reported being familiar with the Freedman and Bernstein studies, [7,27] thus these data were removed from the analysis. No differences in performance on the examination were observed between participants who completed the examination in a proctored versus an un-proctored setting (p = 0.465). Therefore, all responses were included in the analyses.

**Figure 1:**
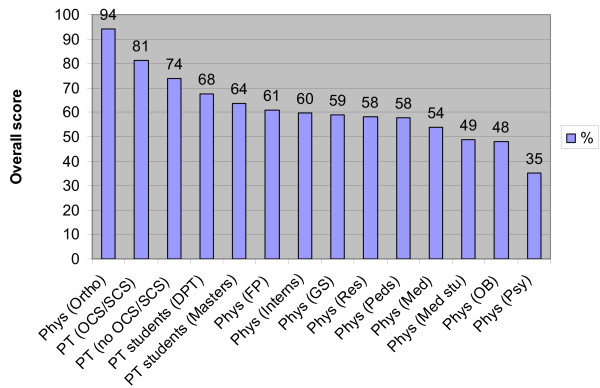
Overall scores on the musculoskeletal knowledge examination among physical therapist students, licensed physical therapists, and previous data using the same examination among physicians. All physician-related data was derived from Matzkin et al,[12] except data for the subgroup of physician interns, which was derived from Freedman and Bernstein[7]. PT = physical therapist, Phys = physician, OCS = Orthopaedic Clinical Specialist, SCS = Sports Clinical Specialist, DPT = doctoral physical therapy program, MPT = master's physical therapy program, Ortho = orthopaedics, Other = anesthesia, emergency medicine. ophthalmology, radiology, and transitional, FP = family practice, GS = general surgery, Res = Resident, Peds = Pediatrics, Med = internal medicine, Med stu = medical student, OB = obstetrics-gynecology, and Psy = psychiatry

Overall scores among the physical therapist students and licensed physical therapists are reported in Figure 1. We did not directly compare the results between physical therapists and physicians using inferential statistics because the data from these groups were derived from unrelated studies. However, the identical examination and similar procedures were used in these studies, thus it is reasonable to discuss our findings in relation to the previous data among physicians. To facilitate this discussion, we superimpose the overall scores among the different physician subgroups with those of the different physical therapist subgroups. This provides a frame of reference for visualizing possible differences in knowledge related to managing musculoskeletal conditions between physical therapists and physicians (Figure 1).

Licensed physical therapists (n = 182) achieved an average score of 75.9%, with an overall passing rate of 67%. Licensed physical therapists who were board-certified achieved significantly higher scores and passing rates than their non board-certified colleagues (Table 2). Physical therapist students (n = 174) achieved an average score of 66.2%, with an overall passing rate of 24%, versus a 19% passing rate among physician interns [7]. Physical therapist students enrolled in programs conferring the doctoral degree achieved significantly higher scores than students enrolled in programs conferring the master's degree, although passing rates were statistically similar (Table 1).

**Table 1:** Performance on the musculoskeletal knowledge examination between physical therapists enrolled in a program that confers a master's vs. a doctoral degree. (Participants were judged to have passed if their score exceeded 73.1%[7].)

**Degree Status (n = 174)**	**Master's (n = 63) (95% CI)**	**Doctoral (n = 111) (95% CI)**	**p-value**
Overall score	63.6 (60.6, 66.6)	67.6 (65.6, 69.6)	.022
Passing rate (Overall score >.731)	.21 (.11, .31)	.26 (.18, .34)	.416

**Table 2:** Performance on the musculoskeletal knowledge examination based on board-certification status. (Participants were judged to have passed if their score exceeded 73.1%[7].)

**Board-certification (OCS and/or SCS) (n = 182)**	**Yes (n = 52) (95% CI)**	**No (n = 130) (95% CI)**	**p-value**
Overall score	81.3 (79.2, 83.4)	73.7 (71.9, 75.5)	<.001
Passing rate	.88 (.80, .97)	.58 (.50, .67)	<.001

## Discussion

Physicians assessed in the study by Freedman and Bernstein had just begun their internship year, [7] and Matzkin et al [12] reported data from medical students, residents, and a variety of physician specialists. Given the spectrum of physician experience levels and specialties represented in previous studies, [7,12] these data offer a compelling reference standard for at least a preliminary discussion related to the preparation of physical therapists versus physicians with respect to managing musculoskeletal conditions. It also seems reasonable to make preliminary general observations about possible differences between physical therapists and physicians since we used the identical examination and administered the examination using similar procedures as those used in the previous studies [7,12].

Figure 1 reveals that both physical therapist students and licensed physical therapists tended to have higher levels of knowledge in managing musculoskeletal conditions than medical students, physician interns and residents, and all physician specialists except for orthopaedists. This trend may seem somewhat intuitive since topics related to managing musculoskeletal conditions are emphasized in physical therapy curricula. However, data were previously lacking to support this contention. It is important to consider that the physician data were derived from unrelated studies, [7,12] thus we discuss our results in general terms in relation to the previous studies among physicians [7,12]. The implication that physical therapists have higher levels of knowledge in managing musculoskeletal conditions than physicians provide impetus for further prospective research in this area.

It could be argued that performance among physical therapist students remains suboptimal, supported by the fact that physical therapist students overall achieved an average score of 66.2%. However, the average score among medical students and interns (the most comparable physician group) was 49% [12] and 60%, [7] respectively. One of the primary curricular areas more heavily emphasized in doctoral physical therapy educational programs is the differential diagnosis of these conditions, a proficiency necessary for competence in more autonomous practice settings such as primary care [30]. Although passing rates were statistically similar, overall scores among physical therapists enrolled in doctoral programs was significantly higher than for master's programs (Table 1). These data provide preliminary evidence that an increased focus on the diagnosis of commonly encountered musculoskeletal conditions and orthopaedic emergencies is occurring in the curricula of doctoral physical therapy programs. However, a threshold of 73.1% was established by orthopaedic program directors as a minimum level of knowledge necessary for competency in musculoskeletal medicine. Given similar passing rates, and in light of increasing availability of direct access care for patients with musculoskeletal conditions, orthopaedic curricula among doctoral physical therapy programs should continue to be enhanced.

Both physicians and physical therapists are at a relative early juncture in their clinical education upon graduation from medical school or physical therapy school, thus they might be expected to have scores below the level established by residency program directors. However, the licensed physical therapists in this study demonstrated higher levels of knowledge in managing musculoskeletal conditions than physical therapist students and all physician subgroups, except for orthopaedists (Figure 1). Licensed physical therapists achieved an average score of 75.9% and an overall passing rate of 67%. This seems to be markedly improved compared to the passing rate amongst all physician subgroups except orthopaedists [12]. Furthermore, most physicians, and with increasing frequency physical therapists, receive graduate medical education in the form of clinical residencies which lead to board certification. In fact, board certification in orthopaedic physical therapy represents the largest area of specialization by physical therapists [31]. One of the key findings from this study was that performance among licensed physical therapists who were board-certified was significantly better when compared to their non board-certified colleagues, lending further credibility to the physical therapist board-certification process, which was not initiated until the 1980s.

Several limitations should be considered. Similar to medical education, physical therapy educational programs do not utilize standardized curricula, thus exposure to didactic and clinical education experiences related to the management of musculoskeletal conditions differs. Physical therapists with a stronger background in this area may have achieved higher scores than with less exposure to an orthopaedic curricula. Content of the examination was also primarily focused on the differential diagnosis of commonly encountered musculoskeletal diagnoses in a primary care setting (ie, fractures and dislocations, low back pain, sciatica, and arthritis) and orthopaedic emergencies that warrant immediate referral to an orthopaedic surgeon or the emergency department (ie, compartment syndrome, hip dislocation, etc.) [7]. Therefore, these data may not be generalizable to other physical therapy practice settings. We invited volunteer physical therapist students and licensed physical therapists to participate, thus the potential for selection bias cannot be excluded. However, physician participants in the study by Matzkin et al [12] were also volunteers, posing a similar limitation that likely mitigates any potential bias in discussing our results in relation to this study. Furthermore, although the examination in the Freedman and Bernstein study [7] was apparently completed by all physicians in the intern class, the examination was only administered to one class [7]. The fact that physical therapist students from a wide variety of programs and licensed physical therapists in geographical locations throughout the country participated in this study increases the generalizability of the findings. Future research could be performed to determine if the results demonstrated among licensed physical therapists in the uniformed services who participated in this study would be similar to the results among a group of civilian physical therapists.

## Conclusion

The results of this study corroborate existing clinical studies demonstrating that physical therapists can provide safe and effective care for patients with musculoskeletal conditions in a direct access setting [21-26]. In comparison to previous studies among physicians, [7,12] physical therapists demonstrated higher levels of knowledge in managing musculoskeletal conditions than medical students, physician interns and residents, and most physician specialists except for orthopaedists. Physical therapist students enrolled in educational programs conferring the doctoral degree achieved higher scores than their peers enrolled in programs conferring the master's degree. Furthermore, licensed physical therapists who were board-certified achieved higher scores and passing rates than their colleagues who were not board-certified. Nevertheless, despite the benefits of early access to physical therapy [14-17] and favorable legislation in most states, [20] the primary barrier to patients receiving physical therapy services without physician referral is that claims are infrequently reimbursed by third party payers. Combined with existing evidence demonstrating that physical therapists are capable of providing safe and effective care for patients with musculoskeletal conditions in a direct access setting at a reduced cost to the healthcare system and employers, the results of this study may have implications for health and public policy decisions regarding the care of patients with musculoskeletal conditions.

## Competing interests

None of the authors of this manuscript have any relevant conflict of interest, financial or otherwise. This study was supported by a grant from the Sports Physical Therapy Section of the American Physical Therapy Association, Inc. The funding organization had no role in the design and conduct of the study, to include data collection; management, analysis, or interpretation of the data. The funding organization was also not involved in the preparation of this manuscript, nor has it been asked to review and/or approve this submission.

## Authors' contributions

JC designed and coordinated the study, performed the statistical analysis, and drafted the manuscript. JW assisted with the study design and drafting of the manuscript. PS developed the web survey instrument and provided oversight for the technical aspects of the survey administration. MP coordinated with the first-professional programs and assisted in the data analysis. TF conceived the idea and assisted with study design and analysis. AD assisted with the study design and acted as a liaison to the program directors. All authors read and approved the final manuscript.

## Disclaimer

The opinions or assertions contained herein are the private views of the authors and are not to be construed as official or as reflecting the views of the U.S. Air Force or Department of Defense.

## Pre-publication history

The pre-publication history for this paper can be accessed here:


